# Role of innate immunity in the development of cancer immunotherapy immune-mediated adverse events

**DOI:** 10.3389/fimmu.2026.1774615

**Published:** 2026-04-15

**Authors:** Álvaro Sierra-Salazar, Yatzil Reyna-Juárez, Beatriz Alcalá-Carmona, Rodrigo Quintana-Tenorio, Jennifer T. Balderas-Miranda, Johan Camacho-Pérez, Jiram Torres-Ruiz

**Affiliations:** Department of Immunology and Rheumatology, Instituto Nacional de Ciencias Médicas y Nutrición Salvador Zubirán, Mexico City, Mexico

**Keywords:** cancer, immune checkpoint inhibitors, immune-related adverse events, immunotherapy, innate immune response

## Abstract

Immune checkpoint inhibitors (ICIs) have transformed modern cancer therapy by restoring antitumor T-cell responses through blockade of immune tolerance pathways such as CTLA-4 and PD-1/PD-L1. However, the same immune activation that underlies their clinical efficacy can also lead to immune-related adverse events (irAEs), a broad spectrum of inflammatory and autoimmune toxicities that may affect virtually any organ system. The incidence and severity of these events vary according to the specific agent, tumor type, and treatment strategy. While irAEs have traditionally been attributed to dysregulated adaptive immunity, emerging evidence highlights a central and previously underappreciated role for innate immune mechanisms. In this review, we integrate the concepts of immunosurveillance and tumor immunoediting to illustrate how innate immunity contributes to both effective antitumor responses and immune-mediated toxicity. We describe how damage-associated signals and tumor microenvironment cues reprogram innate immune populations—including neutrophils, macrophages, dendritic cells, myeloid-derived suppressor cells, and innate lymphoid cells—toward pro-inflammatory or immunosuppressive states that influence therapeutic outcomes and toxicity risk. Finally, emerging biomarkers are highlighted and key knowledge gaps that currently limit the prediction and prevention of irAEs, positioning innate immunity as a critical regulatory axis and a promising target for developing strategies to mitigate toxicity without compromising anticancer efficacy.

## Introduction

The challenges and limitations of conventional cancer treatments have led to the development of new therapeutic strategies that leverage the immune system’s mechanisms for recognizing and eliminating malignant cells. In this context, cancer immunotherapy is understood as a set of therapeutic strategies that intervene in the mechanisms of stimulation, expansion, suppression, or desensitization of the immune system, with the aim of restoring an effective antitumor immune response (1).

Current immunotherapy strategies aim to redirect the immune response against malignant cells using targeted drugs such as monoclonal antibodies, fusion proteins, or small molecules that interact with receptors or ligands on the surface of tumor or immune cells. These are complemented by therapies based on cytokines, oncolytic viruses, antitumor vaccines, and adoptive cell approaches, such as T-cell transfer and CAR-T cell therapies (1, 2).

Immune checkpoints, notably the cytotoxic T-cell-associated protein 4 (CTLA-4) and the programmed cell death protein 1 (PD-1) pathways, maintain the balance between T-cell activation and inhibition to protect the body from harmful immune responses (3). Tumors manipulate these tolerance systems by activating immune checkpoints and inhibiting T cells to evade the antitumor immune response. Immune checkpoint inhibitors (ICIs) have been developed to counteract this immune control, enhancing antitumor immune responses by blocking multiple targets, such as CTLA-4, PD-1, programmed cell death ligand 1 (PD-L1), and lymphocyte activation gene 3 (LAG-3) (3, 4).

Cancer immunotherapy can generate immune-mediated toxicities, called immune-related adverse events (irAEs). Most irAEs are organ-specific and generally occur between 2 and 16 weeks after the start of treatment, depending on the affected body system (5). The time for the development of irAEs also varies according to the mechanism of action: up to nearly the fourth week with ipilimumab, the tenth week with nivolumab, and, in some rare cases, after treatment discontinuation. Furthermore, toxicity does not show a clear association with the administered dose (5). IrAEs also vary according to the type of malignancy and treatment combination. Combined treatments are strongly associated with cutaneous and endocrine toxicities. Furthermore, the incidence and severity of adverse events can vary depending on the treated neoplasm, indicating that the toxicity profile depends on both the type of ICI and the specific tumor context (6).

The incidence of irAEs ranges from 54% to 76%. In pooled series, adverse events have been observed in up to 90% of patients treated with anti-CTLA-4 agents, primarily dermatological, gastrointestinal, and renal toxicities. Approximately 70% of patients treated with anti-PD-1/PD-L1 agents experience arthralgia, pneumonitis, hepatic, and endocrine toxicity, while anti-PD-L1 agents, such as atezolizumab, are mainly associated with endocrine irAEs (3, 6). IrAEs are routinely recorded and classified according to the National Cancer Institute’s Common Terminology Criteria for Adverse Events (CTCAE), which categorizes them from grades 1 to 5, ranging from mild to life-threatening or fatal (7). Cutaneous irAEs are the most frequent, including mild rashes and pruritus, followed by gastrointestinal toxicities such as diarrhea and colitis. Endocrine irAEs, such as hypothyroidism, hyperthyroidism, hypophysitis, and adrenal insufficiency, constitute the third most common group. Other affected systems include the musculoskeletal (mild joint or muscle pain) and the ocular system (dry eye syndrome and uveitis). Less frequent, although potentially serious, are myocarditis, neurotoxicity, myositis, nephritis, and hematological toxicity. Mortality associated with severe irAEs ranges from 10% to 17%, and is exceptionally high in cases of myocarditis, reaching 39.7% (6).

Systemic autoimmune diseases may also be triggered by these treatments. Some cases of systemic lupus erythematosus, polymyalgia rheumatica, and temporal arteritis have been described (8). The most frequent rheumatological manifestations are articular (36%), muscular (34%), granulomatous (6%), and vasculitis (12%) (1). Myalgia has been described in 3% of patients, while myositis occurs in only 0.6% (1). Sicca syndrome has been observed in 5% of patients receiving monotherapy and in 10% of those receiving combined therapy (1). In **Table 1**, we depict the main ICIs, their most frequent indications and the frequency of irAEs.

**Table 1:** Immune-related adverse events (irAEs) associated with immune checkpoint inhibitors (ICIs) (2, 6, 9).

Checkpoint target	Drug	Mechanism	Main clinical indications	Most common immune-related adverse events (irAEs)
CTLA-4	Ipilimumab	CTLA-4 blockade increases co-stimulatory signaling and enhances early T-cell activation and proliferation.	Melanoma (advanced/adjuvant); renal cell carcinoma (in combination regimens); selected MSI-high tumors	Rash/pruritus (30%–50%), immune-mediated colitis (30%–40%; higher incidence and severity vs PD-1/PD-L1 inhibitors), severe diarrhea (10%–15%), hypophysitis (5%–15%; characteristic of CTLA-4 blockade), hepatitis (3%–9%), other endocrinopathies (thyroiditis, adrenal insufficiency), less commonly pneumonitis
PD-1	Nivolumab	PD-1 blockade restores effector T-cell activity	NSCLC, melanoma, renal cell carcinoma, gastroesophageal junction cancer	Rash/pruritus (34%–40%), colitis (19%), severe diarrhea (9%), hypothyroidism (up to 53%), pneumonitis (3%–19%), hepatitis (7%–14%), arthralgia (13%), myocarditis (1%–1.9%)
PD-1	Pembrolizumab	PD-1 blockade restores effector T-cell activity	NSCLC, melanoma, renal cell carcinoma, gastroesophageal cancer, head and neck squamous carcinoma	Rash (34%–40%), colitis (10%–20%), hypothyroidism (42%–53%; up to 56% with CTLA-4 combination), transaminase elevation (7%–14%), pneumonitis (3%–19%), arthralgia (13%), acute kidney injury (2.2%)
PD-1	Camrelizumab	PD-1 blockade restores effector T-cell activity	Hepatocellular carcinoma, NSCLC, esophageal carcinoma	Reactive cutaneous capillary endothelial proliferation (≈80%), hepatotoxicity (7%–14%)
PD-1	Toripalimab	PD-1 blockade restores effector T-cell activity	Nasopharyngeal carcinoma, esophageal squamous carcinoma	Nausea (69%), vomiting (67%), anorexia (53%), constipation (39%), peripheral neuropathy (30%–40%), hypothyroidism (10%–20%)
PD-1	Cemiplimab	PD-1 blockade restores effector T-cell activity	NSCLC, cutaneous squamous cell carcinoma	Arthralgia (18%), myalgia (2–20%), mild colitis (~10%)
PD-1	Serplulimab	PD-1 blockade restores effector T-cell activity	Esophageal squamous carcinoma	Hyperglycemia/insulin-dependent diabetes (~6%), colitis (~10%)
PD-1	Sintilimab	PD-1 blockade restores effector T-cell activity	NSCLC, Hodgkin lymphoma, gastric cancer	Elevated AST (16%), bilirubin elevation (29%), immune-mediated hepatitis (6%–13% monotherapy; up to 38% in combination)
PD-L1	Durvalumab	PD-L1 blockade reduces inhibitory signaling to T cells	Stage III NSCLC, urothelial carcinoma	Rash (~32%), pneumonitis (13%), colitis (10%–20%), hepatotoxicity (7%–14%)
PD-L1	Atezolizumab	PD-L1 blockade reduces inhibitory signaling to T cells	Multiple solid tumors	Transaminase elevation (36%), bilirubin elevation (29%), acute kidney injury (2.2%)
PD-L1	Avelumab	PD-L1 blockade reduces inhibitory signaling to T cells	Merkel cell carcinoma	Diarrhea (62%), nausea (69%), infusion reactions (12%; severe 1.6%)
PD-L1 + VEGFR	Avelumab + Axitinib	PD-L1 blockade + VEGFR tyrosine kinase inhibition (anti-angiogenic)	Advanced renal cell carcinoma	Diarrhea (~62%), infusion reactions (~12%)
PD-1 + CTLA-4	Nivolumab + Ipilimumab	Dual checkpoint blockade enhancing T-cell activation	Renal cell carcinoma, melanoma	Rash (21%), vitiligo (8%), immune colitis (13%–20%), severe diarrhea (~9%), myocarditis
CTLA-4	Tremelimumab	CTLA-4 blockade increases T-cell activation and expansion	Hepatocellular carcinoma	Rash (~32%), colitis (20%–30%), hepatitis (7%–14%)
PD-L1 + CTLA-4	Durvalumab + Tremelimumab	Dual checkpoint blockade	Advanced small-cell lung cancer	Rash (~32%), colitis (15%–25%)
PD-L1 + VEGF	Atezolizumab + Bevacizumab	PD-L1 blockade + VEGF inhibition	Hepatocellular carcinoma	Elevated AST (~36%), bilirubin elevation (~29%)
PD-L1	Adebrelimab	PD-L1 blockade restores T-cell antitumor activity	Small-cell lung cancer	Neutropenia (95%), leukopenia (94%), anemia (85%), thrombocytopenia (82%)

HCC, hepatocellular carcinoma; NSCLC, non-small cell lung cancer; MSI-H, Microsatellite instability–High; SCLC, small-cell lung cancer; VEGF, vascular endothelial growth factor; ALT, alanine aminotransferase; AST, aspartate aminotransferase.

The underlying pathogenic mechanisms of irAEs are likely multifactorial and include aberrant activation of autoreactive and tumor-reactive T lymphocytes, which can react against antigens shared by the tumor and normal tissue, as well as hyperactivation of B lymphocytes and increased production of autoantibodies (2). Nonetheless, lymphocytes are not the only players in the induction of irAEs, as three types of inflammatory infiltrates are observed: neutrophilic, lymphocytic, or combined (9). Therefore, components of innate immunity may interact with lymphocytes to enhance the autoimmune response in irAEs, but this has not been thoroughly studied. In the following sections, we depict the anti-tumor innate immune response and its potential role in the development of irAEs.

## Innate immune response in cancer

The immune system plays a crucial role in the antitumor immune defense. Immunosurveillance refers to the continuous process in which immune cells recognize and eliminate genetically or phenotypically altered cells that may become malignant, thereby preserving tissue integrity and homeostasis. Over time, this concept evolved into the tumor immunoediting model, including three successive phases: elimination, equilibrium, and escape. Tumor cells that resist immune surveillance can adapt, evade immune control, and promote their growth and migration to other tissues (10). This phenomenon is further influenced by the fact that the immune system does not always exert a protective role; it can also contribute to tumor progression by switching the immunogenic phenotype of cancer cells (11).

Immunosurveillance starts with the activation of innate immunity, which represents the first barrier against carcinogenesis. Following the onset of cancer-associated intrinsic mutations, deregulated proliferation, cellular stress, hypoxia, and tissue necrosis promote the release of damage-associated molecular patterns (DAMPs) such as ATP, HMGB1, and calreticulin (12–14), thereby triggering an inflammatory response within the tumor microenvironment (TME).

These DAMP signals are sensed by intracellular pattern-recognition receptors that translate tissue damage into inflammatory signaling. One group of receptors that plays a key role in initiating antitumor immune responses is the NOD-like receptor (NLR) family, a class of cytosolic pattern-recognition receptors involved in sensing intracellular danger signals. Among them, the NLRP3 inflammasome is the best characterized and functions as a multiprotein complex capable of integrating signals derived from pathogen-associated molecular patterns (PAMPs) and DAMPs generated during tumor development, including extracellular ATP, mitochondrial reactive oxygen species (ROS), oxidized mitochondrial DNA, potassium efflux, lysosomal damage, and metabolic stress associated with malignant transformation (15–17).

Inflammasome activation profoundly shapes the interplay between innate and adaptive immunity in cancer. On the one hand, NLRP3 signaling may contribute to antitumor immunosurveillance by promoting IL-18-dependent activation of natural killer (NK) cells and cytotoxic T lymphocytes, as well as enhancing dendritic cell-mediated priming of adaptive immune responses (16, 18). On the other hand, persistent inflammasome activation can promote tumor progression by sustaining chronic inflammation, inducing IL-1β-dependent recruitment of immunosuppressive myeloid populations such as tumor-associated macrophages (TAMs) and myeloid-derived suppressor cells (MDSCs), and facilitating angiogenesis, epithelial–mesenchymal transition, and metastatic dissemination (17–19). Taken together, NLRP3 inflammasome signaling is increasingly recognized as a context-dependent regulator of tumor immunity, capable of either enhancing immunosurveillance or promoting tumor growth depending on the cellular and inflammatory landscape of the tumor microenvironment (15–19).

The innate immune response in cancer plays a dual role: it seeks to limit tumor progression through the recognition and elimination of malignant cells, but when inflammation becomes persistent and unresolved, the sustained oxidative stress and continuous tissue damage foster the accumulation of mutations, reduce tumor immunogenicity, and drive the immunosuppressive polarization of microenvironmental cells. As a result, these mechanisms enable tumor cells to evade immune control and advance disease progression (10, 11, 20).

TAMs represent one of the most abundant immune populations within solid tumors and participate in virtually all stages of tumor development, from the initial detection of transformed cells to the invasion and metastasis processes (21–24). Their remarkable plasticity depends on the tissue, cellular metabolism, and signals derived from the TME, which has challenged the traditional M1/M2 dichotomy and led to the recognition of a far more complex functional spectrum (23–26). Tissue-resident macrophages (TRMs), mostly derived from embryonic progenitors, rapidly respond to tissue damage or stress (20, 22–24, 27). In the context of cancer, DAMPs released by necrotic or hypoxic cells activate TRMs through sterile inflammatory pathways (12, 13, 26), promoting cytokine release and the activation of effector functions that limit tumor growth. Moreover, TRMs facilitate the recruitment of additional immune cells (27). As the disease progresses, TAMs can be reprogrammed toward phenotypes that favor immune evasion. The production of TGF-β by tumor cells and macrophages, along with Th2-type cytokines (IL-4, IL-13), induces VEGF expression and the recruitment of Tie2^+^ monocytes, a pro-angiogenic subset that promotes irregular and functionally defective neovascularization (27–30). In parallel, the secretion of matrix metalloprotease (MMP)-9, IL-10, and TGF-β, along with the expression of arginase-1 (ARG1), shapes an immunosuppressive and profibrotic microenvironment that inhibits T-cell cytotoxicity and favors metastatic dissemination through extracellular matrix remodeling (27–30). Additional mechanisms, such as Vascular Cell Adhesion Molecule-1 (VCAM-1)-mediated signaling or the CD47–Signal-regulatory protein (SIRP)α pathway, contribute to tumor cell survival within the microenvironment (28, 30, 31). Collectively, through these processes TAMs promote cancer progression (21, 27–30).

Following TRM activation by tumor-derived DAMPs, the secretion of IL-8 and granulocyte-colony stimulating factor (G-CSF) promotes neutrophil infiltration into the TME. Initially, these cells complement the antitumor response through their phagocytic activity and release of antimicrobial granules. However, as the disease progresses, their function switches toward supporting tumor progression (28). A central mechanism of this transition is the formation of neutrophil extracellular traps (NETs), web-like structures composed of DNA, histones, and granular enzymes that remodel the TME. NETs promote chronic inflammation and act as scaffolds that trap circulating tumor cells. Together with neutrophil-derived MMP-9, they facilitate extravasation and metastasis (32, 33). In addition, NET-derived DNA can be recognized by the coiled-coil domain containing 25 (CCDC25) receptor on tumor cells, activating signaling pathways that enhance cell motility and drive metastatic migration, particularly to the liver (34). Moreover, NETs create physical and chemical barriers that limit T and NK cell infiltration, increase resistance to immune checkpoint inhibition, and are associated with post-treatment relapse. In preclinical models, NETosis inhibition, either through Peptidyl Arginine Deaminase 4 (PAD4) blockade or DNase I treatment, reduces metastasis and improves immunotherapy responsiveness (32, 34–37). Collectively, NETs and MMP-9 derived from neutrophils and macrophages, and VEGF secretion represent central mediators of angiogenesis, immunosuppression, and metastatic dissemination, consolidating their role as determinants of immune evasion (20, 21, 28).

Innate lymphoid cells (ILCs) also have dual roles in tumor immunity. Derived from the same progenitor as T lymphocytes but lacking a T-cell receptor (TCR), ILCs respond to microenvironmental signals through activating and inhibitory receptors. They are classified into cytotoxic cells, natural killer (NK), and helper-like subsets (ILC1, ILC2, and ILC3), whose functions depend on the prevailing cytokine microenvironment (38).

NK cells exhibit cytotoxicity toward tumor or infected cells without requiring MHC restriction or expression of stress-related receptors such as major histocompatibility complex (MHC) Class I polypeptide-related sequence (MIC) A and B. Their activation depends on the balance between activating and inhibitory receptors, such as NKG2D, NKp30, NKp44, NKp46, CD226, and CD16 (FcγRIIIa), which mediate antibody-dependent cellular cytotoxicity (ADCC). Inhibitory receptors include killer cell immunoglobulin-like receptor (KIR), NKG2A/CD94, PD-1, T cell immunoreceptor with Ig and ITIM domains (TIGIT), TIM-3, and LAG-3 (39, 40). Once activated, NK cells release perforin and granzymes A/B, inducing apoptosis through intrinsic and extrinsic pathways. They also secrete interferon (IFN)-γ and TNF-α, which promote conventional type 1 dendritic cell (cDC1) recruitment and CD8^+^ T-cell activation (38, 40). However, immunosuppressive factors present in the tumor microenvironment can limit these functions. In particular, TGF-β has been shown to reduce the surface expression of the activating receptor NKG2D on NK cells and cytotoxic lymphocytes, impairing their ability to recognize and eliminate cells expressing NKG2D ligands such as MICA, MICB, and members of the UL-16 binding protein (ULBP) family (41, 42). Mechanistically, this effect is mediated through TGF-β–induced interference with the transcriptional and post-transcriptional regulation of activating receptors such as NKG2D and NKp30 via SMAD-dependent transcriptional programs, ultimately reducing NK cell activation, cytotoxicity, and IFN-γ production (41, 43). Prolonged exposure to TGF-β may even induce transdifferentiation of NK cells into non-cytotoxic ILC1s, characterized by loss of perforin and expression of CD49a, thereby contributing to angiogenesis and tissue remodeling (38–40).

Regarding ILCs, ILC1s differentiate under stimulation with IL-12, IL-15, and IL-18, which induce the expression of the transcription factor T-bet. Their principal role in the antitumor response is the production of IFN-γ and tumor necrosis factor (TNF)-α, cytokines that promote CD8^+^ T-cell activation, dendritic cell maturation, and enhance macrophage and neutrophil phagocytosis. During early cancer stages, these functions contribute to immunosurveillance and limit metastasis. However, within a TME dominated by TGF-β and other immunosuppressive signals, ILC1s lose cytotoxic capacity and acquire a regulatory phenotype that favors tumor progression (38). ILC2s, induced by GATA-3 polarization by IL-33, IL-25, and thymic stromal lymphopoietin (TSLP), produce IL-5, IL-9, IL-13, granulocyte-macrophage colony-stimulating factor (GM-CSF), and amphiregulin (AREG). Under IL-33 activation, ILC2s strengthen antitumor immunity by promoting CD103^+^ DC and CD8^+^ T-cell recruitment. Conversely, in IL-13-, TGF-β-, or PGE_2_-rich microenvironments, they expand MDSCs and regulatory T cells (Tregs), stimulate angiogenesis, and contribute to tumor progression (38). ILC3s, characterized by RORγt expression following polarization by IL-6 and TGF-β, produce IL-22, IL-17, TNF-α, and IL-8. These cytokines can support antitumor immunity by inducing endothelial activation and tertiary lymphoid structure formation, but persistent exposure to tumor cells promotes a proangiogenic and immunosuppressive phenotype (38).

MDSCs are one of the principal mediators of tumor-associated immune regulation. Initially described as immature myeloid cells, single-cell transcriptomic and cytometric studies have revealed that they represent transient functional states rather than fixed lineages (44, 45). Their expansion occurs during persistent inflammation and hypoxia, driven by cytokines such as IL-6, GM-CSF, G-CSF, and prostaglandins, which activate STAT3, NF-κB, and C/EBPβ pathways, skewing myeloid differentiation toward immunosuppressive phenotypes (44, 46). Two main subtypes are recognized: monocytic (M)-MDSCs are CD14^+^/HLA-DR^low^, produce IL-10, TGF-β, and IDO1, and express PD-L1, ARG1, and COX2, thereby inhibiting CD8^+^ T-cell and NK-cell activation (44, 47). Polymorphonuclear (PMN)-MDSCs, phenotypically similar to CD15^+^ neutrophils, secrete reactive oxygen species (ROS), nitric oxide (NO), MMP-9, and S100A8/A9, promoting angiogenesis and tissue remodeling (45, 48). Both subtypes cooperate to suppress adaptive immunity, block dendritic cell (DC) maturation, downregulate NKG2D signaling in NK cells, and promote the expansion of Tregs and M2-like macrophages (46, 49). MDSCs act through several mechanisms: metabolic suppression via ARG1, iNOS, and IDO1, which depletes essential amino acids; secretion of IL-10 and TGF-β, which inhibits the expression of costimulatory molecules; expression of PD-L1, Galectin-9, and B7-H4, which represses effector functions of lymphocytes; and the production of exosomal miR-146a/155, which reprograms other immune cells toward tolerogenic profiles (44, 50). In murine models of melanoma, lung, and pancreatic cancer, inhibition of MDSC function via CXCR2 or STAT3 antagonists restores CD8^+^ infiltration and enhances immunotherapy efficacy. Increased circulating MDSCs correlate with poor prognosis and therapy resistance across multiple cancers (45, 51).

After the initial innate immune response, activation of adaptive immunity largely depends on DC function. In proinflammatory environments, conventional type 1 dendritic cells (cDC1s) respond to tumor-derived DAMPs, promoting their maturation and antigen-processing capacity (12, 52, 53). These cells secrete IL-12, type I interferons (IFN-I), and the chemokines CXCL9 and CXCL10, which are essential for the differentiation of cytotoxic CD8^+^ T cells through cross-presentation of antigens via MHC-I and for polarization toward a Th1 response (52–56). In *Batf3^-^/^-^* murine models, the absence of cDC1s reduces the efficacy of anti-PD-1/PD-L1 therapies, highlighting their indispensable function in antitumor immunotherapy (57). However, the TME can reprogram DC function. The secretion of VEGF, IL-10, TGF-β, and PGE_2_ blocks DC maturation and limits their ability to activate T cells (21, 53). Under these conditions, a distinct population known as mature DCs enriched in immunoregulatory molecules (mregDCs) emerges. These cells acquire a tolerogenic phenotype and localize in perilymphatic regions within the tumor. Characterized by the expression of LAMP3, CCR7, PD-L1, and IDO1, mregDCs engage in close crosstalk with Tregs, reinforcing local immunosuppression (58). This interaction reduces DC migration to draining lymph nodes, thereby limiting antigen presentation and CD8^+^ T-cell activation (58). Furthermore, secretion of CCL17 and CCL22 recruits CCR4^+^ Tregs, while CD80/CD86–CTLA-4 interactions potentiate their suppressive activity (58). In multiple human tumors—including colorectal and lung cancers—the coexpression of FOXP3, LAMP3, and CCL22 has been correlated with poor immunotherapy response and unfavorable prognosis, establishing mregDCs as critical checkpoints of tumor immune evasion (58, 59).

**Figure 1:**
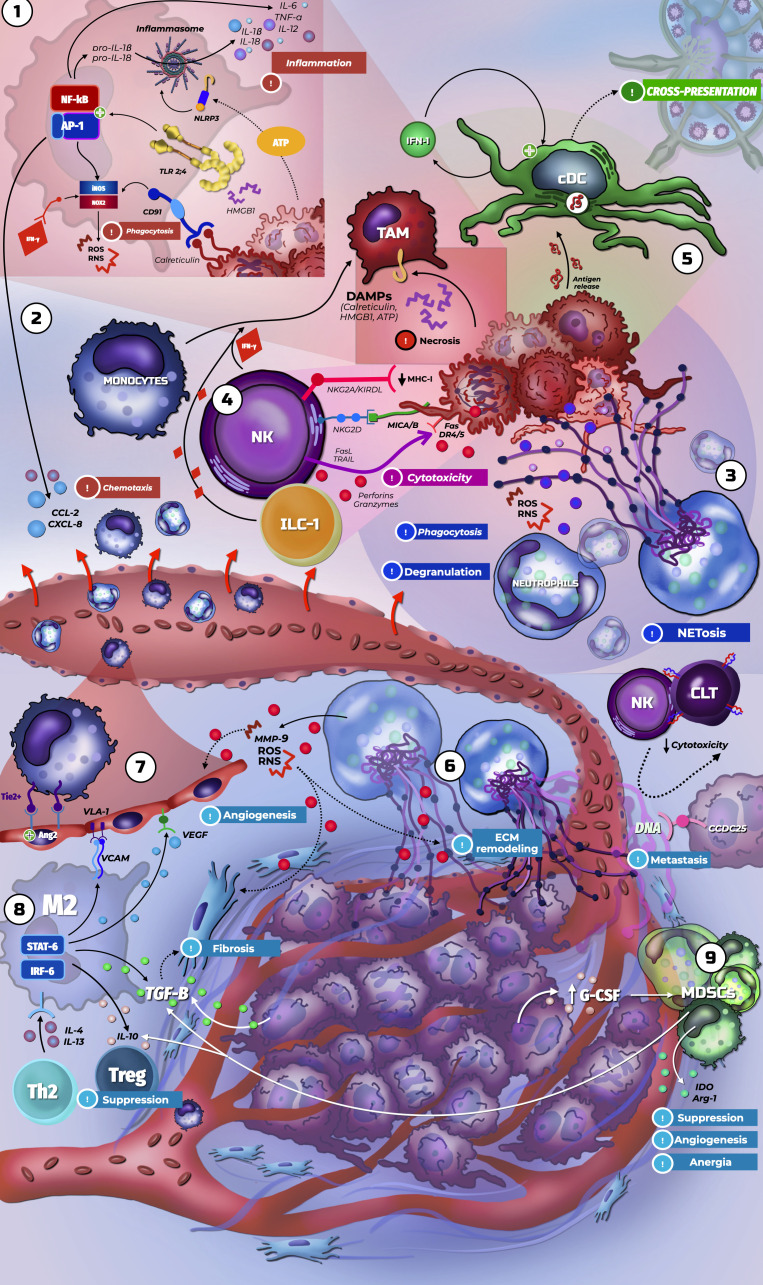
Innate immune response against cancer.

In conclusion, innate immunity not only represents the first barrier against cancer but also serves as a regulatory axis that can dictate the success or failure of the antitumor response. The plasticity of myeloid and innate immune cells determines whether the TME becomes oriented toward malignant cell elimination or tumor protection. Understanding the transitions between these functional states is therefore essential for designing therapeutic strategies that restore immunosurveillance and enhance the efficacy of modern cancer therapies. In **Figure 1**, we represent the innate antitumor immune response. ICIs also modulate innate immunity, and an expanded innate immune response contributes to the development of irAEs, as shown in the following section.

## Role of innate immunity in the development of irAEs

As first responders of innate immunity, neutrophils comprise diverse subpopulations with context-dependent phenotypes. Cancer and inflammation can drive emergency granulopoiesis and the release of immature neutrophils, including low-density granulocytes (LDGs) with unique functions (60). Clinical studies have identified a high neutrophil-to-lymphocyte ratio (NLR) as a risk factor for grade 4–5 irAEs and fatal toxicities (61). The NLR is widely considered a surrogate marker of systemic inflammation and immune imbalance, reflecting the predominance of neutrophil-driven inflammatory responses relative to lymphocyte-mediated immune regulatory mechanisms (62, 63). Neutrophilia is associated with increased release of pro-inflammatory mediators, reactive oxygen species, and the production of NETs, all of which can amplify inflammatory responses and promote tissue injury (62, 64, 65). At the same time, a relative reduction in circulating lymphocytes may indicate diminished immune regulatory capacity and reduced control of excessive immune activation. In the context of immune checkpoint blockade, this imbalance may predispose patients to exaggerated immune responses once key inhibitory pathways such as PD-1/PD-L1 or CTLA-4 are blocked. Consequently, a high NLR may reflect a pre-existing pro-inflammatory immune state that favors dysregulated immune activation following ICI therapy, thereby increasing susceptibility to severe irAEs and organ-specific inflammatory toxicities (62–64).

Tumor-associated neutrophils (TANs) and PMN-MDSCs, often expanded in cancer patients, may likewise alter immune homeostasis. For example, cancer progression is accompanied by a profound alteration in neutrophil output and function, which can interfere with ICI outcomes (66). In patients with prostate cancer treated with the combination of anti-CTLA-4 and anti-PD-L1 agents, an increased macrophage and neutrophil transcriptional signature was found in bone marrow biopsies, suggesting that immunotherapy can promote granulopoiesis (67).

Tissue-infiltrating neutrophils are frequently observed in organ-specific irAEs. Neutrophils accumulate in subcorneal aggregates in PD-1 induced psoriasis (68), suggesting an effector pathogenic function of granulocytes in this complication. In ICI-induced colitis, neutrophils are markedly expanded in intestinal lesions (69). These infiltrating neutrophils release proteolytic enzymes (e.g., elastase, MMPs) and ROS that directly injure epithelial barriers and the surrounding stroma (69, 70). Neutrophils can also amplify inflammation by secreting pro-inflammatory cytokines (such as IL-1β) and chemokines (such as CXCL8/IL-8), which recruit additional neutrophils and lymphocytes, creating a feed-forward loop of tissue inflammation.

A key pathway linking adaptive and innate responses in irAEs is the Th17–neutrophil axis. Checkpoint blockade can provoke exuberant IL-17 production by CD4^+^ Th17 cells in tissues (71). IL-17, in turn, induces epithelial and stromal cells to secrete neutrophil-attracting chemokines, driving robust neutrophil recruitment. Notably, patients with high baseline serum IL-17 levels have an increased risk of severe ICI-related colitis (72), underscoring the role of neutrophil-rich inflammation in gastrointestinal toxicity. Upstream inflammatory signals such as IL-6 and IFN-γ further reinforce this cascade by promoting Th17 differentiation and neutrophil mobilization (73).

Neutrophils can also mediate irAEs through antibody-dependent mechanisms. Many ICI therapies are monoclonal IgG antibodies, and neutrophils express Fcγ receptors capable of recognizing IgG-coated targets. In inflammatory settings, TNF-α and IFN-γ induce clustering of high-affinity FcγRI on neutrophils (priming), enhancing their binding to immune complexes (74). Consequently, off-target binding of ICIs to normal tissues can trigger neutrophil activation via FcγRs (75). An example of this mechanism is the depletion of regulatory T cells by anti-CTLA-4 antibodies. Anti-CTLA-4 can engage FcγRs on myeloid cells, leading to ADCC against CTLA-4+ Treg cells (74). Similarly, immune complexes formed by ICIs can activate neutrophils. A recent study demonstrated that repeated anti-PD-L1 antibody administration in tumor-bearing mice induced an IgG-mediated anaphylactic reaction in which neutrophils were central effectors (76). In that model, tumor-associated neutrophils and macrophages released massive amounts of platelet-activating factor (PAF) upon FcγR engagement, precipitating fatal anaphylaxis (76). Importantly, depletion of neutrophils protected the mice from shock (76). This finding highlights how neutrophils, when stimulated through Fc–FcγR interactions, can drive systemic irAEs.

Another potential contributor to irAEs is the production of NETs. In the context of cancer immunotherapy, sterile triggers such as DAMPs and high cytokine levels may induce NETosis. Excess NET formation has two major immunopathological consequences relevant to irAEs: direct tissue damage and the breaking of self-tolerance. NET structures contain a concentrated mix of proteases (elastase, cathepsin G, proteinase-3) and oxidant enzymes (myeloperoxidase) that can degrade extracellular matrices and inflamed tissues. For example, NET-derived elastase can degrade endothelial tight junctions, contributing to vasculitis and capillary leakage. NETs can also occlude small vessels, and their pro-thrombotic nature may underlie the thromboembolic events or microvascular complications occasionally seen with ICIs (77). NETs are also a potential source of autoantigens, providing a bridge between innate and adaptive immunity (78). Therefore, the role of NETs in the development of irAEs requires further investigation.

Monocytes and macrophages are key players in the development of irAEs. In patients with different types of neoplasms treated with distinct ICIs, a higher baseline absolute monocyte count has been widely proposed as a biomarker for the future development of irAEs, with a cutoff value >0.29 k/μl (odds ratio (OR) 2.34, 95% confidence interval (CI) 1.06–5.15, P = 0.03) (79, 80). Monocytes are expanded in the peripheral blood of patients with non-small cell lung cancer who develop ICI-associated myocarditis, especially in those who died due to this complication (81).

Immunophenotyping studies have helped gain a better understanding of the monocyte profiles associated with the development of immune toxicity. For instance, previous studies have shown an expansion of proinflammatory intermediate monocytes (CD14^+^ CD16^+^) and increased expression of the activation marker HLA-DR in patients with thyroiditis induced by PD-1/PD-L1 inhibitors (82, 83). Likewise, analysis of ICI-induced lichenoid dermatitis lesions demonstrated marked infiltration of this monocyte subset, along with a transcriptional upregulation of Toll-like receptor pathways (84). *In vitro* assays with synovial fluid mononuclear cells (SFMCs) have shown that pembrolizumab not only induces the intermediate monocyte subset but also enhances their secretion of MCP-1, TNFα, IL-10, IL-12p70, IL-13, IFNγ, IL-2, and IL-4, but not IL-6 or IL-1 (85). Nonetheless, this functional effect has not been proven to be preserved at a systemic level in patients with ICI-induced inflammatory arthritis or other immune adverse effects. Other authors have reported an expansion of peripheral classical monocytes (CD14^++^ CD16^−^), showing a positive correlation with ALT levels in ICI-induced hepatitis (r = 0.52, P = 0.02) (86). Additionally, compared with patients receiving ICIs without irAEs, classical monocytes from patients with ICI-induced hepatitis showed higher expression of the tissue-homing receptor CCR2 and the macrophage activation marker CD163 (86).

Other novel monocyte phenotypes, such as the CD14−MC−CCL3 subset, have been reported to expand in patients who develop ICI-induced hepatitis after anti-PD-1 treatment. This subset was characterized by the expression of IL1β, CCL3, and CXCL8, and the highest IFN-γ score among the monocyte subpopulations (87). Nevertheless, whether this cell subset is a result or a driver of the dysregulated proinflammatory response found in irAEs still needs to be determined.

In patients receiving ICIs, macrophages acquire a highly plastic dual role, simultaneously favoring antitumor responses and inducing uncontrolled inflammation and systemic autoimmune toxicity (88). TAMs normally tend to display an immunosuppressive (M2-like) phenotype, which limits T-cell cytotoxicity through the production of IL-10 and TGF-β and the expression of PD-1, CD47–SIRPα, CTLA-4, V-domain Ig suppressor of T-cell activation (VISTA), and Siglec-10 (89–91). Blockade of these interactions with ICIs partially reverses this suppression and drives a proinflammatory antitumor M1-like state, induced by IFN-γ and characterized by increased phagocytosis, secretion of IL-12, TNF-α, IL-1, and IL-6, and overexpression of costimulatory molecules such as CD80/CD86, MHC-II, and HLA-DR (92). The induction of an M1 phenotype is dependent on the enhanced expression of the NLRP3 inflammasome, as has been demonstrated in animal models of ICI-induced myocarditis (81).

Rather than acting as an initiating event, inflammasome activation appears to function primarily as an amplifier of tissue inflammation, driven by the accumulation of damage-associated molecular patterns (DAMPs) and pro-inflammatory cytokines generated in response to ICI therapy. This process promotes caspase-1–dependent maturation of IL-1β and IL-18, enhances immune cell recruitment, and sustains tissue injury through self-reinforcing inflammatory circuits (93–95).

This mechanism is particularly relevant in ICI-associated myocarditis. Experimental studies have demonstrated increased myocardial expression of NLRP3, accompanied by elevated levels of IL-1β, IL-6, and cardiac injury biomarkers such as troponin T and NT-proBNP (95). In addition, activation of caspase-1, gasdermin D, and pyroptosis has been observed in cardiomyocytes, suggesting that NLRP3-mediated inflammatory cell death directly contributes to myocardial injury. Importantly, pharmacological inhibition of this pathway attenuates inflammation and cardiac damage, supporting the inflammasome as a relevant therapeutic target (94). This beneficial shift for antitumor activity also breaks peripheral tolerance and predisposes to the development of irAEs.

Macrophages are part of the inflammatory infiltrate of the target tissues of irAEs, including the skin, colon, lung, liver, heart, pancreas, joints, thyroid, muscle, and stomach, and reproduce histological and transcriptional patterns observed in autoimmune diseases (67, 96–99). In immune-mediated colitis, a CD68^+^/CD86^+^ infiltrate predominates, with a high production of IL-1β, IL-6, and TNF-α, as well as expression of CXCL1, CXCL9, and CXCL10 (CXCR3 ligands), which contribute to epithelial disruption, recruitment of T lymphocytes and neutrophils, and the stimulation of the IL-23/Th17 axis (100–102). Studies of bronchoalveolar lavage in ICI-associated pneumonitis have described an increased secretion of CXCL10 (IP-10) and other cytokines/chemokines associated with CD11b^+^ myeloid/monocyte–macrophage populations; these data support a role of macrophages and a Th1 response in ICI-induced pulmonary inflammation (103, 104). In addition, histopathological analyses have described the characteristic presence of foamy alveolar macrophages, located in alveolar spaces and interstitial septa, reflecting phagocytic and inflammatory activation linked to alveolar damage (105).

Macrophages are key players in the development of ICI-induced myocarditis (106). Checkpoints protect the myocardium against excessive inflammation by limiting excessive T-cell activation and M1-like macrophage polarization (107). Histopathological studies and murine models of ICI-associated myocarditis have shown mixed infiltrates of CD8^+^ T cells and CD68^+^ macrophages, with expansion of the inflammatory CCR2^+^ subpopulations that mediate myocardial necroinflammation and tissue remodeling (106, 108, 109). Recent clinical data confirm a significant increase in IL-6, IL-8, CXCL9, and CXCL10 in patients with ICI-associated myocarditis, correlating with clinical severity and elevations of troponin and NT-proBNP. This pattern reinforces the notion of an inflammatory signature dominated by activated macrophages and the IFN-γ/CXCR3 axis (110). Important gaps remain in the pathophysiology of these events, as not all irAEs display the same macrophage profile, and hybrid M1/M2 phenotypes exist due to cellular plasticity, exhibiting both inflammatory and anti-inflammatory functions (111, 112).

As the main activators of adaptive immunity, DCs are at the core of T-cell activation. Under physiological conditions, they maintain the balance between immunity and tolerance. However, ICIs disrupt this balance by enhancing DC maturation to a proinflammatory phenotype, thereby contributing to the development of irAEs (89, 113). The main DC subpopulations in cancer include cDC1 and cDC2, monocyte-derived DCs (moDCs), plasmacytoid DCs (pDCs), and mature and mregDCs, each having specific functions (92). cDC1 (CD141^+^) promote cross-presentation and activation of cytotoxic CD8^+^ T lymphocytes through IL-12 and CXCL9/10. cDC2 (CD1c^+^) favor CD4^+^ T-cell differentiation toward Th1/Th17 phenotypes via IL-6 and IL-23, while pDCs (CD123^+^) are the main producers of IFN-I (IFN-α/β) in response to DNA/RNA signals through the TLR7/9–MyD88 pathway (92, 114). Under checkpoint blockade conditions, these pathways are exacerbated, driving excessive DC activation and an exaggerated effector T-cell response.

The expression of PD-L1, PD-L2, and CD80/CD86 on DCs is critical for peripheral homeostasis. In the absence of PD-1/PD-L1 and CTLA-4 signaling, DCs exhibit sustained maturation, with increased CD83, IL-12, and MHC-II expression, and a reduction in tolerogenic mechanisms such as IDO activity and IL-10 secretion (115–117). This phenotypic shift reinforces the priming of autoreactive T lymphocytes and promotes organ-specific inflammation (118). In peripheral venous blood, increased levels of cDC1 (119) and pDCs (120) have been reported in patients who developed irAEs. Histologically, infiltration of cDCs and pDCs has been documented in pneumonitis (121), myocarditis (122), and various cutaneous manifestations (123, 124). Nonetheless, a decreased number of pDCs and cDCs was found in peripheral blood of patients with immune-related myocarditis (122). In patients with ICI-induced psoriasis, massive infiltration of the skin by CD11c^+^ DCs has been observed (68), whereas monocyte-derived DCs are expanded in peripheral blood of patients with ICI-induced colitis (125). The role of DCs as orchestrators of irAEs requires further investigation.

Regarding the soluble mediators, innate immune cells are a source of cytokines, chemokines, and IFNs, which are differentially expressed across irAEs. IFNs are essential for the therapeutic efficacy of ICI (126), but their excessive activation constitutes one of the main molecular bases of irAEs (127). Activation of the IFN pathways is fundamental in the pathogenesis of irAEs. Blockade of PD-1/PD-L1 enhances JAK–STAT signaling, increasing macrophage sensitivity to IFN-γ (128). In addition, overexpression of type I and II IFN signaling components has been observed in ICI-induced colitis (125). IFNs induce the transcription of interferon-stimulated genes (ISGs), thereby potentiating tissue inflammation. In parallel, IFN-I (α/β), produced by DCs and macrophages via TLR7/9 or cGAS–STING pathways, reinforce MHC-I/II expression and autoantigen presentation. An elevated IFN-score has been described in patients with severe irAEs (129, 130). IFN-γ amplifies the inflammatory circuit by stimulating the JAK–STAT1 pathway in myeloid cells, inducing secretion of IL-6, TNF-α, and CXCL9/10 (131, 132). This IFN-γ–STAT1–CXCL9/10 axis has been observed in biopsies of ICI-induced colitis, pneumonitis, and hepatitis, and is associated with intense tissue inflammation and steroid refractoriness (133–135). Moreover, nivolumab-induced gastritis is characterized by intense IFN-γ production by epithelial cells (67). The role of IFN-III (λ) is particularly recognized in epithelial tissues such as the intestine, lung, and liver, where they contribute to barrier inflammation and persistence of irAEs in mucosal organs (87). Cytokines and chemokines are key mediators of cell communication that coordinate immune responses and regulate functions such as cell proliferation, differentiation, and survival within the TME (136). In patients with irAEs, marked overexpression of CXCL9 and CXCL10, as well as increased activation of the TNF-α pathway, contributes to an exacerbated inflammatory response (129). Also in ICI-induced myositis, overexpression of the IL-6 pathway has been reported, and patients with ICI-induced DM show an intense type I IFN signature (99). Other irAEs, such as PD-1-induced psoriasis, are characterized by the production of IL17A, IL-23, IFN-γ, IL-22, IL-6, and TNF-α (68), indicating that different cytokines and chemokines may be differentially expressed in irAEs, which supports their use as biomarkers and potential therapeutic targets.

The role of other innate immune cells and complement in the development of irAEs has been less explored. Expansion of MDSCs has been widely associated with poor treatment outcomes and prognosis in diverse malignancies (137). Although the functional properties of MDSCs are directly counteracted by ICIs, their potential role in the development of irAEs remains poorly understood. In patients with stage III and IV melanoma treated with nivolumab or pembrolizumab ± ipilimumab, Lepper et al. found no differences in the frequency of M-MDSCs nor in their expression of PD-L1 and CD73 according to the presence of irAEs. However, after immunosuppressive therapy, patients with irAEs showed an increase in circulating M-MDSCs along with Tregs (138). Although not thoroughly investigated, ICIs have the ability to alter the proportions and phenotypes of ILCs in peripheral blood of patients with metastatic melanoma (139). Transcriptomic analyses of ICI-induced myocarditis have shown an infiltration of the myocardium by T/NK cells (122). Likewise, ICI-induced colitis shows an NK transcriptional signature (125). In patients with irAEs, there is a baseline reduction of NK cells, specifically the CD56^dim^ CD16^+^ subset (129). On the other hand, ICI-induced complement-mediated thrombotic microangiopathies (CM-TMA) have been reported (140). This phenomenon has been associated with complement dysregulation, evidenced by decreased C3 and increased CH50, suggesting excessive activation of the terminal pathway.

**Figure 2:**
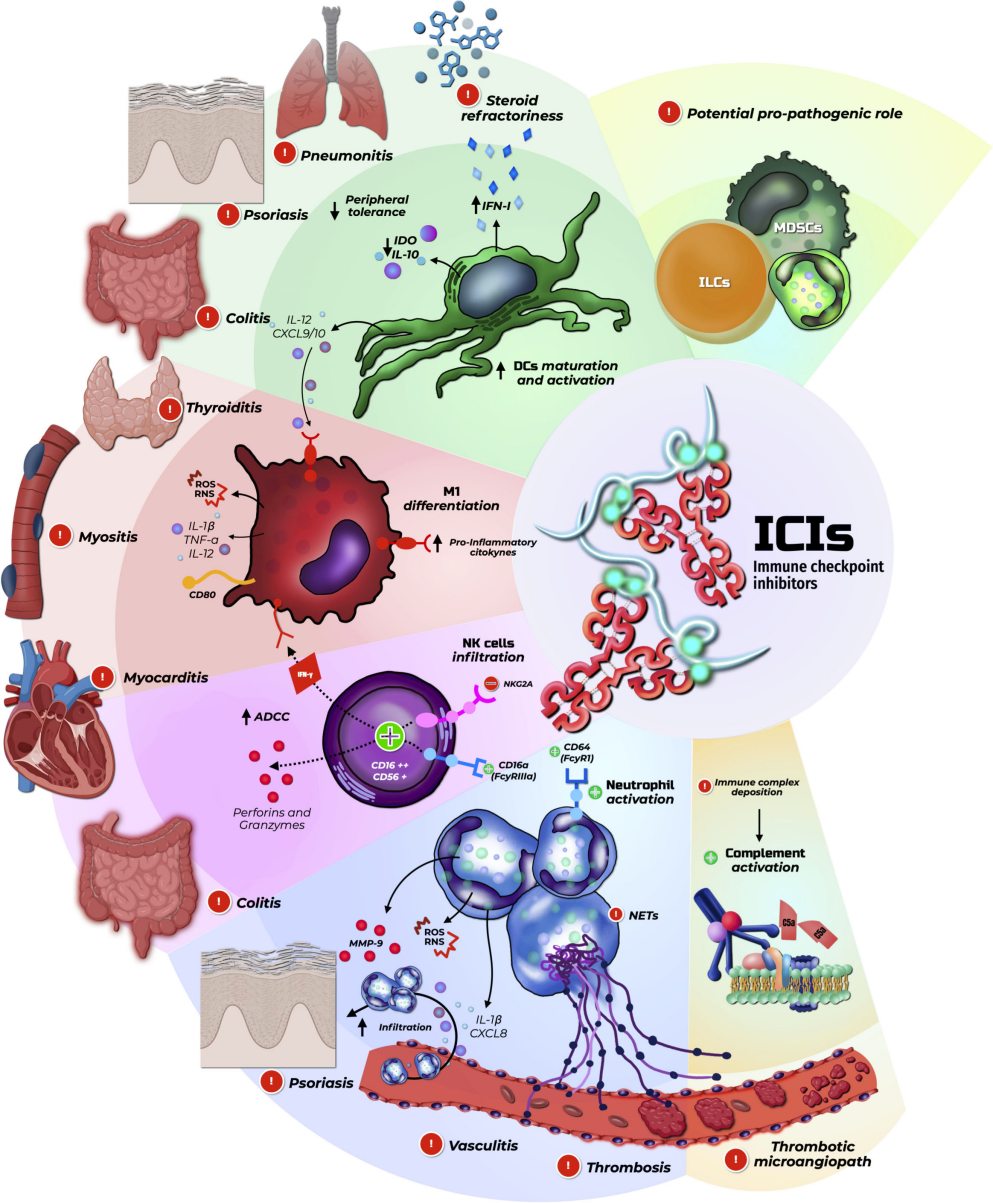
Role of innate immunity in the development of irAEs. Created with BioRender.com.

In **Figure 2**, we provide a graphical abstract summarizing the current evidence on the role of innate immunity in the development of irAEs.

## Conclusion

This review reframes the understanding of immune-related adverse events by positioning innate immunity as a central driver of immunotherapy-induced toxicity, rather than a secondary participant. Immune checkpoint blockade not only enhances adaptive immune responses but also disrupts innate immune homeostasis, thereby becoming a critical determinant of the initiation and propagation of tissue damage induced by ICIs. Importantly, the same innate immune mechanisms that sustain tumor immunosurveillance can, under conditions of sustained activation, promote the transition toward pathological inflammation and autoimmunity. Innate-derived biomarkers—including circulating myeloid subsets, the neutrophil-to-lymphocyte ratio, cytokine signatures, and interferon-associated transcriptional programs—offer promising tools to anticipate toxicity and refine clinical decision-making. At the same time, selective modulation of innate immune pathways, such as IL-6, TNF-α, inflammasome signaling, or myeloid cell recruitment, emerges as a rational strategy to mitigate toxicity without compromising antitumor efficacy, although achieving this balance remains a major therapeutic challenge. Ultimately, integrating innate immune profiling into clinical practice may enable a shift from reactive management toward precision-based strategies focused on prevention and individualized treatment of irAEs, thereby optimizing both the safety and the transformative potential of cancer immunotherapy.

Resident macrophages recognize tumor-derived DAMPs (calreticulin, HMGB1, ATP), triggering PRR activation and the release of pro-inflammatory mediators that initiate immune cell recruitment and differentiation required for effective immunosurveillance. 2. CCL2 and CXCL8 production drives the infiltration of monocytes and neutrophils, which exert context-dependent pro- and anti-tumor functions. 3. Neutrophils mediate antitumor activity through ROS generation, phagocytosis, degranulation (defensins, metalloproteinases), and the formation of neutrophil extracellular traps (NETs). 4. Innate lymphoid cells enhance antigen-presenting cell maturation and phagocytic activity via IFN-γ. NK cells detect MHC loss, stress ligands (e.g., MICA), or IgG-opsonized targets and induce apoptosis through granule-mediated (perforin, granzymes) or death receptor–mediated (FasL, TRAIL) mechanisms. 5. Dendritic cells capture tumor-derived antigens and perform cross-presentation, linking innate and adaptive immunity. 6. NETs also promote tumor progression by inducing inflammation and extracellular matrix remodeling. Cancer cell recognition of NET-derived DNA via CCDC25 enhances migration and metastasis, while NETs suppress NK- and CTL-mediated cytotoxicity. 7. Angiogenesis is promoted by granulocyte-derived MMP-9 and ROS, increased Tie2+ monocytes, and VEGF production, collectively supporting neovascularization and tumor expansion. 8. Tumor-associated macrophages polarize toward an M2 phenotype with reduced phagocytic capacity and increased TGF-β secretion, driving extracellular matrix deposition and immunosuppression through Treg induction. 9. Chronic inflammation and tissue damage facilitate the expansion of myeloid-derived suppressor cells (MDSCs), which suppress lymphocyte activation and further promote angiogenesis.

Immune checkpoint inhibitors (ICIs) promote sustained activation of innate immune cells and, through the reduction of peripheral immune tolerance, favor the development of immune-related adverse events. This disinhibition leads to persistent activation of dendritic cells and macrophages, amplification of interferon signaling via the JAK/STAT pathway, and enhanced autoantigen presentation. Fcγ receptor–dependent mechanisms further potentiate NK cell and neutrophil activation, driving antibody-dependent cellular cytotoxicity, degranulation, reactive oxygen species release, and neutrophil extracellular trap formation. Collectively, these processes result in sterile tissue injury, vascular damage, and organ-specific inflammation, clinically manifesting as colitis, pneumonitis, dermatitis, myocarditis, and vasculitis, and are intrinsically linked to the antitumor efficacy of ICIs.
